# Impact of coronavirus disease 2019 on pulmonary function in early convalescence phase

**DOI:** 10.1186/s12931-020-01429-6

**Published:** 2020-06-29

**Authors:** Yiying Huang, Cuiyan Tan, Jian Wu, Meizhu Chen, Zhenguo Wang, Liyun Luo, Xiaorong Zhou, Xinran Liu, Xiaoling Huang, Shican Yuan, Chaolin Chen, Fen Gao, Jin Huang, Hong Shan, Jing Liu

**Affiliations:** 1 Department of Pulmonary and Critical Care Medicine, The Fifth Affiliated Hospital of Sun Yat-sen University, 52 East Meihua Rd, Zhuhai City, 519000 China; 2 Guangdong Provincial Key Laboratory of Biomedical Imaging, The Fifth Affiliated Hospital of Sun Yat-sen University, 52 East Meihua Rd, Zhuhai City, 519000 China; 3 Department of Cardiovascular Medicine, The Fifth Affiliated Hospital of Sun Yat-sen University, Zhuhai, China

**Keywords:** COVID-19, Early convalescence, Lung function, Respiratory muscle strength

## Abstract

**Objective:**

This study investigated the influence of Coronavirus Disease 2019 (COVID-19) on lung function in early convalescence phase.

**Methods:**

A retrospective study of COVID-19 patients at the Fifth Affiliated Hospital of Sun Yat-sen University were conducted, with serial assessments including lung volumes (TLC), spirometry (FVC, FEV1), lung diffusing capacity for carbon monoxide (DLCO),respiratory muscle strength, 6-min walking distance (6MWD) and high resolution CT being collected at 30 days after discharged.

**Results:**

Fifty-seven patients completed the serial assessments. There were 40 non-severe cases and 17 severe cases. Thirty-one patients (54.3%) had abnormal CT findings. Abnormalities were detected in the pulmonary function tests in 43 (75.4%) of the patients. Six (10.5%), 5(8.7%), 25(43.8%) 7(12.3%), and 30 (52.6%) patients had FVC, FEV1, FEV1/FVC ratio, TLC, and DLCO values less than 80% of predicted values, respectively. 28 (49.1%) and 13 (22.8%) patients had PImax and PEmax values less than 80% of the corresponding predicted values. Compared with non-severe cases, severe patients showed higher incidence of DLCO impairment (75.6%vs42.5%, *p* = 0.019), higher lung total severity score (TSS) and R20, and significantly lower percentage of predicted TLC and 6MWD. No significant correlation between TSS and pulmonary function parameters was found during follow-up visit.

**Conclusion:**

Impaired diffusing-capacity, lower respiratory muscle strength, and lung imaging abnormalities were detected in more than half of the COVID-19 patients in early convalescence phase. Compared with non-severe cases, severe patients had a higher incidence of DLCO impairment and encountered more TLC decrease and 6MWD decline.

## Background

Coronavirus Disease 2019 (COVID-19) is a new and highly contagious respiratory disease caused by severe acute respiratory syndrome coronavirus 2 (SARS-CoV-2), which presented a risk of infection from human to human [1]. The current outbreak of COVID-19 has caused a global pandemic. As of 7 June, 2020, there were 6,663,304 confirmed cases and 392,802 confirmed deaths globally. It might progress rapidly, and some patients developed respiratory failure early in the disease. The knowledge about COVID-19, including clinical manifestations, pathogenesis, even treatment came from research and observation during the acute infection period [2, 3]. In China, the vast majority of the patients had been successfully discharged. Until now, no study have reported early prognosis in relation to the degree of lung injury and rehabilitation in patients with COVID-19. Retrospective study showed that many patients had imaging abnormalities when discharged, a few patients even had pulmonary fibrosis. Lung function damage of patients with COVID-19 in early convalescence phase deserves attention. In order to have a more comprehensive understanding of the possible clinical outcomes of COVID-19, we conducted a retrospective study involving 57 discharged but undergoing rehabilitation COVID-19 patients. Serial lung function, lung imaging examination and exercise capacity were examined at 30 days after discharged. In addition, we compared severe patients with non-severe patients by outcome parameters.

## Materials and methods

### Patient selection

This is a follow up study of COVID-19 patients at 30 days after discharged from our hospital. From January 17, 2020 to March 1, 2020, a total of 103 COVID-19 patients were admitted to the Fifth Affiliated Hospital of Sun Yat-sen University. The diagnosis of COVID-19 was based on the CDC criteria. All patients had laboratory-confirmed SARS-CoV-2 infection by real-time reverse transcription polymerase chain reaction (RT-PCR) or next-generation sequencing. They all reached uniform discharge standard issued by the National Health Commission of China and had been released from the hospital over 1 month. In 30 days after discharged, patients were eligible to participate in the study if they were over 18 years of age. Patients with a previous history of pulmonary resection, neurological disease, or mental illness were excluded from our study. We obtained written informed consent from the patients before pulmonary function testing. This study was approved by the institutional ethics committee of the Fifth Affiliated Hospital of Sun Yat-sen University.

### Classification

We retrospectively analyzed the medical records of these patients, and divided them into non-severe and severe groups according to the severity of the disease. Patients would be defined as severe cases if satisfied any of the following criteria: shortness of breath, RR ≥ 30 times per minute; blood oxygen saturation ≤ 93% in resting state; partial arterial oxygen pressure (PaO2)/ fraction of inspiration O2 (Fi02) ≤ 300mmmHg;respiratory failure requires mechanical ventilation; shock occurred or combined with other organ failure required ICU monitoring and treatment. Otherwise were mild cases.

### Lung imaging acquisition and CT quantitative evaluation

All subjects underwent high resolution spiral CT (SOMATOM Definition Flash Siemens; Erlangen, Germany) scans in the supine position during end-inspiration. Images were reconstructed at 1.0 mm slice thickness, with 1 mm increment, 512 mm × 512 mm. The images were assessed by two radiologists, both of whom were blinded to the clinical information. We used the same method as Michael et al., to quantify pulmonary inflammation severity [4–6]. Briefly, each of the five lung lobes was assessed for degree of involvement and classified as none (0%), minimal (1–25%), mild (26–50%), moderate (51–75%), or severe (76–100%). No involvement corresponded to a lobe score of 0, minimal involvement to a lobe score of 1, mild involvement to a lobe score of 2, moderate involvement to a lobe score of 3, and severe involvement to a lobe score of 4.An overall lung “total severity score” was reached by summing the five lobe scores (range of possible scores, 0–20). All CT scores were independently performed by two respiratory doctors. Agreement was reached by consensus.

### 6 min walk test

Six min walk test (6MWT) is an exercise test that evaluates the functional status which is relevant to daily activities of patients with cardiopulmonary disease. The walking distance is closely related to gender, age and height, conventionally need a hierarchical analysis according to the above parameters. However, the sample size of our study was small which was not suitable for stratified analysis based on age, gender and height. So we estimated the walking distance of healthy people of the same gender, age and height according to reference equations for the 6MWT in healthy adults [7]. Then we calculated the ratio of measured value of the patients to the predicted value of the healthy person in fair condition. By comparing the ratio of two groups we could see whether there was difference in 6MWD between non-severe and severe COVID-19 patients.

### Pulmonary function test and respiratory muscle strength measurement

Each subject underwent a standard pulmonary function test (Master Screen, Jaeger, German). Recorded parameters include: total lung volume (TLC), forced vital capacity (FVC), residual volume (RV), forced expiratory volume in the first second (FEV1), maximum expiratory flow rate (MMEF75/25), FEV1 / FVC ratio, and diffusing capacity of the lung for carbon monoxide (DLCO). Impuse oscillation system (IOS) was used to measure airway viscosity resistance at an oscillation frequency of 5 Hz(R5), and central airway resistance at an oscillation frequency of 20 Hz (R20). Mouth pressure gauges can measure the maximum static inspiratory pressure (PImax) or maximum static expiratory pressure (PEmax) through a flanged cigarette holder. All subjects used this simple method to gauge inspiratory and expiratory muscle strength. The spirometry, DLCO, and respiratory muscle strength measurements were expressed as a percentage of predicted normal values.

To protect lung function laboratory staff, lung function tests were performed in a room with negative pressure device. Staff wore personal protective equipment, including N95 respirators, protective glasses, gloves and gowns. In addition, each patient used disposable virus and bacterial filters during the test.

### Statistics

Statistical analysis was performed using Statistical Package for Social Science (SPSS) Version 13.0. Measurement data was expressed as mean ± standard deviation. Continuous variables were compared using independent-sample t test, whereas the rank sum test was used for nonparametric data. Comparison of proportion was evaluated by Chi-square test. Spearman correlation test was used to detect the correlations between lung function and lung total severity score. All statistical tests were two tailed. Statistical significance was taken as p<0.05.

## Results

### Characteristics of the enrolled COVID-19 patients

**Fig. 1:**
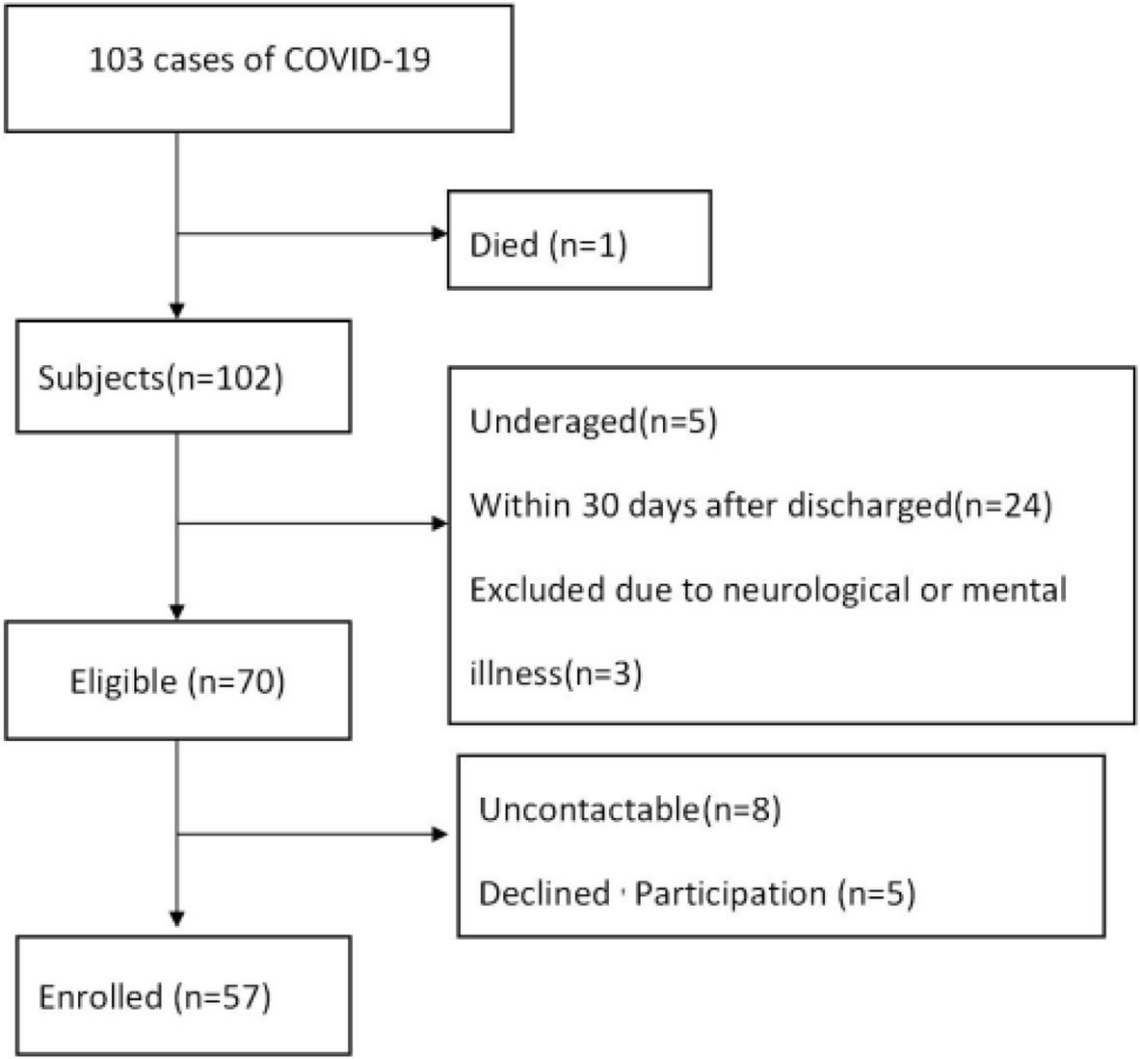
Enrollment of COVID-19 patients in Early Convalescence

This study evaluated a total of 102 patients. Five patients were excluded for underage. Twenty-four patients were not included as it was less than 30 days after discharge. Three patients were excluded due to neurological or mental illness. In addition, eight patients had been out of contact. At last, 57 patients had been included and completed the serial assessments in the study (Fig. 1). There were 26 men and 31 women with a mean age of 46.72 ± 13.78 years (age range, 19 to 71 years),the mean body mass index was 23.99 ± 3.55 kg / m2.Among the 57 subjects, 46 (80.7%) had a history of direct contact with Wuhan, Hubei. Nine patients(15.7%)had a history of smoking. Twenty-one patients (36.8%) had preexisting medical illness. The four most common preexisting illnesses were hypertension (11 patients), diabetes (four patients), malignant tumor (three patients) and cardiovascular disease (three patients). All of these conditions were either healed, or stable and well controlled at the time of testing during the study. No patient was reported having chronic respiratory diseases.

Among all subjects, seventeen were severe cases (29.8%), forty were non-severe cases (70.2%). There were mainly male patients(70.6%)in the severe group, and the average age of patients was older compared with non-severe cases. The mean Pao2/Fio2 ratio among severe cases was significantly lower than non-severe cases (198.47[SD, 97.04]; 355.51[SD, 37.23], P<0.001). Meanwhile, severe cases had higher serum lactate dehydrogenase (LDH), C-reactive protein (CRP) peaks and lower lymphocyte count compared with non-severe cases. But there was no significant difference in the values of white blood cells, creatine kinase (CK), lactic acid peaks and length of hospitalization between the two groups (Table 1).

**Table 1:** Demographic and clinical characteristic of COVID-19 patients

Characteristic	Total (*n*=57)	Severe (*n*=17)	Non-severe (*n*=40)	*p* Value
Age, year	46.72 (13.78)	52.53 (13.30)	44.25 (13.3)	0.031
Male gender, No	26 (45.6%)	12 (70.6%)	14 (35.0%)	0.014
BMI, kg/m2	23.99 (3.55)	25.54 (3.43)	23.33 (3.42)	0.103
Pre-existing medical illness	21 (36.8%)	7 (41.2%)	14 (35%)	0.658
LOS, days	20.89 (7.22)	20 (16-24)	19 (15-24)	0.834
WBC, ×10^9/L	5.01 (1.50)	4.47 (1.35)	5.24 (1.52)	0.076
lymphocyte count, ×10^9/L	1.60 (0.55)	1.30 (0.35)	1.72 (0.58)	0.008*
CRP, mg/dL	9.69 (13.77)	22.65 (18.19)	4.18 (5.66)	<0.001*
LDH, U/L	175.47 (43.60)	201.94 (43.96)	164.22 (38.76)	0.002*
CK, U/L	91.95 (118.16)	133.18 (209.4)	74.42 (31.69)	0.235
Lactic acid, mmol/L	1.59 (0.61)	1.51 (0.65)	1.62 (0.59)	0.511
PaO_2_ to FiO_2_ ratio, mmHg	308.67 (94.40)	198.47 (97.04)	355.51 (37.23)	<0.001*
TSS on the worst chest CT scan	4.28 (4.26)	8.59 (4.15)	2.45 (2.73)	<0.001*
TSS on chest CT on the 14th day after discharge	1.75 (2.23)	3.94 (2.33)	0.83 (1.39)	<0.001*
glucocorticoids use	16 (28.1%)	11 (64.7%)	5 (12.5%)	<0.001*
Total methyprednisolone dosage, mg	213.75 (323.87)	289.09 (370.4)	48.0 (17.89)	0.019*

Values are expressed as mean (SD)

*Statistically significant

### Lung function tests and respiratory muscle strength

Table 2 presents the results of pulmonary function tests and respiratory muscle strength among COVID-19 patients. During follow-up at 1 month after hospital discharge, there were 30 individuals (52.6%) with abnormal diffusion capacity among the 57 patients participating in our study. According to the ATS recommendations for evaluating respiratory impairment [8], twenty-six patients (86.7%) had mild impairment of DLCO, while the other four (13.3%) had moderate impairment. There was significant difference in impaired diffusing-capacity between the two groups, which accounted for 42.5% in non-severe cases, and 75.6% in severe cases, respectively (*p* < 0.05, Table 3).

**Table 2:** Results of pulmonary function tests and respiratory muscle strength among COVID-19 patients

Parameter	Total (*n*=57)	Severe (*n*=17)	Non-severe (*n*=40)	*p* Value
FVC (% of predicted)	100.96 (15.93)	95.92 (19.59)	103.10 (13.83)	0.12
FEV1 (% of predicted)	97.89 (14.91)	93.93 (16.79)	99.57 (13.92)	0.194
FEV1/FVC(%)	81.22 (6.13)	80.58 (4.88)	81.49 (6.62)	0.614
TLC (% of predicted)	93.94 (12.75)	88.72 (16.20)	96.22 (10.35)	0.048*
RV (% of predicted)	90.68 (28.08)	86.57 (23.96)	92.47 (29.82)	0.327
DLCO (% of predicted)	78.38 (13.59)	74.14 (18.85)	80.12 (10.56)	0.139
Raw(% of predicted)	105.38 (31.38)	99.46 (26.32)	108.03 (33.38)	0.524
R5(% of predicted)	126.64 (29.45)	118.75 (29.98)	130.00 (28.96)	0.072
R20(% of predicted)	132.76 (30.95)	120.15 (31.46)	138.12 (29.50)	0.024*
Pi max (% of predicted)	76.16 (24.28)	80.49 (29.24)	74.26 (21.93)	0.382
Pe max (% of predicted)	102.73 (32.68)	98.00 (27.11)	104.80 (34.96)	0.637
6MWD, m	561.97 (45.29)	517.43 (44.55)	573.52 (38.38)	0.012*
6MWD (% predicted)	94.61 (6.55)	88.46 (7.61)	96.20 (5.31)	0.011*

Values are shown as mean (SD) severe vs non-severe with *p* values

*Statistically significant

**Table 3:** The abnormal rate of pulmonary parameters and respiratory muscle strength between severe cases and mild cases

Characteristic	Total (*n*=57)	Severe (*n*=17)	Non-severe (*n*=40)	chisq	*p* value
FEV1 < 80% of pred	5 (8.8)	3 (17.6)	2 (5.0)	1.066	0.302
FEV1 ≥ 80% of pred	52 (91.2)	14 (82.4)	38 (95.0)		
FVC < 80% of pred	6 (10.5)	4 (23.5)	2 (5.0)	2.604	0.107
FVC ≥ 80% of pred	51 (89.5)	13 (76.5)	38 (95.0)		
FEV1 / FVC < 80%	25 (43.9)	9 (52.9)	16 (40.0)	0.811	0.368
FEV1 / FVC ≥ 80%	32 (56.1)	8 (47.1)	24 (60.0)		
TLC < 80% of pred	7 (12.3)	4 (23.5)	3 (7.5)	1.552	0.213
TLC ≥ 80% of pred	50 (87.7)	13 (76.5)	37 (92.5)		
DLCO < 80% of pred	30 (52.6)	13 (76.5)	17 (42.5)	5.522	0.019^*^
DLCO ≥ 80% of pred	27 (43.4)	4 (23.5)	23 (57.5)		
R5 ≥ 150 of pred	8 (14.0)	2 (11.8)	6 (15.0)	0.103	0.554
R5 < 150 of pred	49 (86.0)	15 (88.2)	34 (85.0)		
R20 ≥ 150 of pred	10 (17.5)	3 (17.6)	7 (17.5)	0.000	0.631
R20 < 150 of pred	47 (82.4)	14 (82.4)	33 (82.5)		
PImax < 80% of pred	28 (49.1)	9 (52.9)	21 (52.5)	0.001	0.976
PImax ≥ 80% of pred	29 (50.9)	8 (47.1)	19 (47.5)		
PEmax < 80% of pred	13 (22.8)	4 (23.5)	9 (22.5)	0.007	0.592
PEmax ≥ 80% of pred	44 (77.2)	13 (76.5)	31 (77.5)		

*pred* predicted

*Statistically significant

The group means of forced expiratory volume in 1 s (FEV1), static lung volumes were within normal limits (>80% predicted). However, several cases of abnormalities in FVC, FEV1, and FEV1/FVC ratio were detected. Five patients (8.7%) had mild impairment of FVC, one (1.8%) had moderate impairment of FVC, 5 (8.7%) had mild impairment of FEV1, and 25 (43.9%) had mild impairment of FEV1/FVC. There were 8 patients (14.0%) and 10 patients (17.5%) had increased R5 and R20 more than 150% of the predicted value, respectively. Up to 12.2%(*n* = 7) of patients had reduction in parameters of lung volume (TLC) at 1 month. Among them, 6 had mild impairment, one had moderate impairment. TLC declined more significantly in severe cases (*p* = 0.048). There was no difference in FVC, FEV1, and FEV1/FVC between the two groups. Table 4 shows the detailed pulmonary function data of all 57 subjects. The majority of the impairment in FEV1 and FVC suggests a restrictive abnormality. One patient without history of asthma had obstructive abnormality with a FEV1/FVC ratio<70% predicted (up to 72% after bronchodilation), who had significant history of cigarette smoking. Although no complained of symptoms of asthma, one other patient had a significant bronchodilator response with increments of FEV1>200 ml after inhalation of salbutamol.

**Table 4:** Clinical and Pulmonary Function Data of COVID-19 Patients (*n* = 57)

Patient No	Age, yr	Clinical type	FVC	FEV 1	FEV 1 % FVC	DLCO SB	TLC	R 5	R20
1	41	2	85.80	90.40	90.57	77.90	92.60	99.10	103.40
2	36	2	114.20	114.50	86.71	79.40	110.90	113.60	153.50
3	29	2	103.80	105.00	87.81	72.20	100.00	112.50	102.30
4	65	1	90.60	88.50	77.09	71.50	81.00	122.70	117.60
5	52	1	75.90	75.90	80.77	82.00	69.90	197.70	195.70
6	36	1	75.70	80.40	89.21	68.00	81.80	117.10	117.20
7	56	2	72.00	74.60	87.38	64.20	70.70	144.20	167.10
8	33	2	114.50	114.10	83.82	71.70	98.40	91.20	112.50
9	54	1	94.30	89.30	79.95	60.60	83.50	108.60	101.30
10	42	1	84.60	83.60	81.67	92.50	78.70	172.50	171.00
11	38	2	102.70	94.90	79.88	77.20	93.70	97.50	99.60
12	33	2	86.80	90.00	89.78	67.50	94.60	125.10	137.60
13	69	1	88.00	86.00	75.27	48.60	77.40	113.00	112.70
14	37	2	74.40	62.00	71.88	71.80	77.30	146.50	167.40
15	20	2	85.70	73.70	79.04	78.60	82.00	121.60	159.60
16	71	1	91.90	88.00	79.50	49.20	89.30	109.60	90.60
17	36	1	83.00	83.20	82.98	72.80	79.40	76.10	85.90
18	33	2	95.00	98.10	89.22	72.80	100.30	225.20	202.70
19	37	2	96.60	108.00	96.25	70.00	108.20	178.30	189.60
20	63	2	93.30	84.90	76.31	72.40	91.00	115.30	93.90
21	37	2	118.20	93.70	68.55	80.10	103.80	134.20	107.80
22	60	1	59.70	64.30	84.89	50.10	53.20	111.50	117.90
23	56	2	103.40	102.00	79.22	70.90	90.60	114.40	138.70
24	32	1	86.80	85.30	85.33	53.30	106.40	86.90	86.50
25	36	2	101.00	100.40	85.89	64.00	91.70	161.50	148.70
26	62	1	100.90	92.90	77.07	72.90	97.70	168.50	128.40
27	39	2	106.90	93.30	72.55	66.80	92.90	78.20	86.40
28	29	2	113.30	100.50	74.41	74.70	108.10	110.10	135.70
29	22	2	75.60	85.70	98.91	65.40	122.90	107.60	103.80
30	25	2	98.60	99.20	85.31	79.00	92.40	139.60	158.70
31	64	2	111.60	106.40	79.28	73.80	100.40	127.00	131.80
32	32	2	116.30	106.00	78.92	68.60	106.60	240.10	183.70
33	56	2	111.60	105.30	80.22	79.10	98.80	147.20	134.90
34	62	1	134.90	128.60	75.45	119.60	117.00	114.30	88.60
35	39	2	109.10	92.10	73.04	89.20	106.20	126.90	125.50
36	53	1	104.00	99.90	78.96	87.00	96.30	142.70	70.20
37	29	2	96.90	84.10	75.20	92.50	96.70	126.50	100.90
38	22	1	111.70	111.40	77.85	81.50	91.70	150.50	73.10
39	25	2	106.50	98.20	78.22	84.40	91.90	87.90	80.10
40	66	2	89.20	88.60	77.03	96.10	80.30	119.80	98.30
41	56	2	133.60	125.60	78.00	98.10	103.40	131.30	111.60
42	64	1	127.90	117.30	75.16	81.20	114.80	139.30	110.40
43	56	1	121.20	112.10	76.98	81.30	102.50	127.10	103.30
44	36	2	106.90	114.20	88.53	80.00	91.80	121.90	120.70
45	49	2	111.10	108.30	82.66	82.90	108.80	129.50	144.80
46	38	1	99.50	110.10	91.75	82.10	87.50	94.90	115.30
47	39	2	90.80	87.20	79.87	107.50	81.20	151.30	159.50
48	44	2	106.60	105.20	80.86	96.50	96.80	142.90	153.00
49	29	2	96.00	91.20	79.98	92.30	91.60	118.70	139.70
50	59	2	109.50	103.60	79.77	92.70	93.80	127.20	152.50
51	55	2	114.00	124.00	86.51	91.50	96.00	117.20	121.00
52	63	2	109.50	104.10	79.15	80.30	92.00	128.70	135.70
53	57	2	116.80	114.60	82.91	89.80	99.10	90.60	115.00
54	59	2	96.20	91.30	79.66	86.00	96.30	137.20	143.70
55	55	2	129.30	124.60	81.32	82.80	113.80	116.40	142.10
56	65	2	101.20	107.90	83.10	84.80	86.00	103.80	99.50
57	63	2	119.50	115.20	81.19	85.30	95.10	137.50	145.70

Values given as % predicted, unless otherwise indicated. Clinical type, 1 for severe, 2 for non-severe

More than half of the subjects had impairment in respiratory muscle strength. There were 28 patients (49.1%) and 13 patients (22.8%) had Pimax and Pemax values less than 80% of the predicted value, respectively.13 patients had moderate impairment of respiratory muscle strength, of whom 11 were non-severe cases (Table 4). When grouped by the administration of steroid, no statistical significance was found in respiratory muscle strength between the glucocorticoid group and the regular group (Table 5).

**Table 5:** Results of pulmonary function tests and respiratory muscle strength among COVID-19 patients between glucocorticoid group and the regular groups

Parameter	All patients (*n*=57)	GC group (*n*=16)	Regular group (*n*=41)	*p* Value
FVC (% of predicted)	100.96 (15.93)	97.25 (18.69)	102.40 (14.72)	0.414
FEV1 (% of predicted)	97.89 (14.91)	94.35 (15.40)	99.27 (14.68)	0.279
FEV1 / FVC (%)	81.22 (6.13)	80.74 (4.68)	81.40 (6.65)	0.804
TLC (% of predicted)	93.94 (12.75)	90.15 (16.01)	95.45 (11.06)	0.323
RV (% of predicted)	90.68 (28.08)	85.49 (19.01)	92.76 (30.95)	0.593
DLCO (% of predicted	78.38 (13.59)	74.67 (14.37)	79.78 (13.20)	0.657
Raw (% of predicted)	105.38 (31.38)	96.02 (25.81)	109.22 (32.93)	0.214
R5 (% of predicted)	126.64 (29.45)	119.66 (30.62)	129.37 (28.91)	0.127
R20 (% of predicted)	132.76 (30.95)	123.51 (31.99)	136.37 (30.15)	0.106
Pimax (% of predicted)	76.16 (24.28)	85.21 (26.54)	72.53 (22.65)	0.059
Pemax (% of predicted)	102.73 (32.68)	104.22 (28.03)	102.14 (34.68)	0.479

Values given as % predicted, unless otherwise indicated

*GC* glucocorticoid

### Chest radiographs and correlations with lung function

**Fig. 2:**
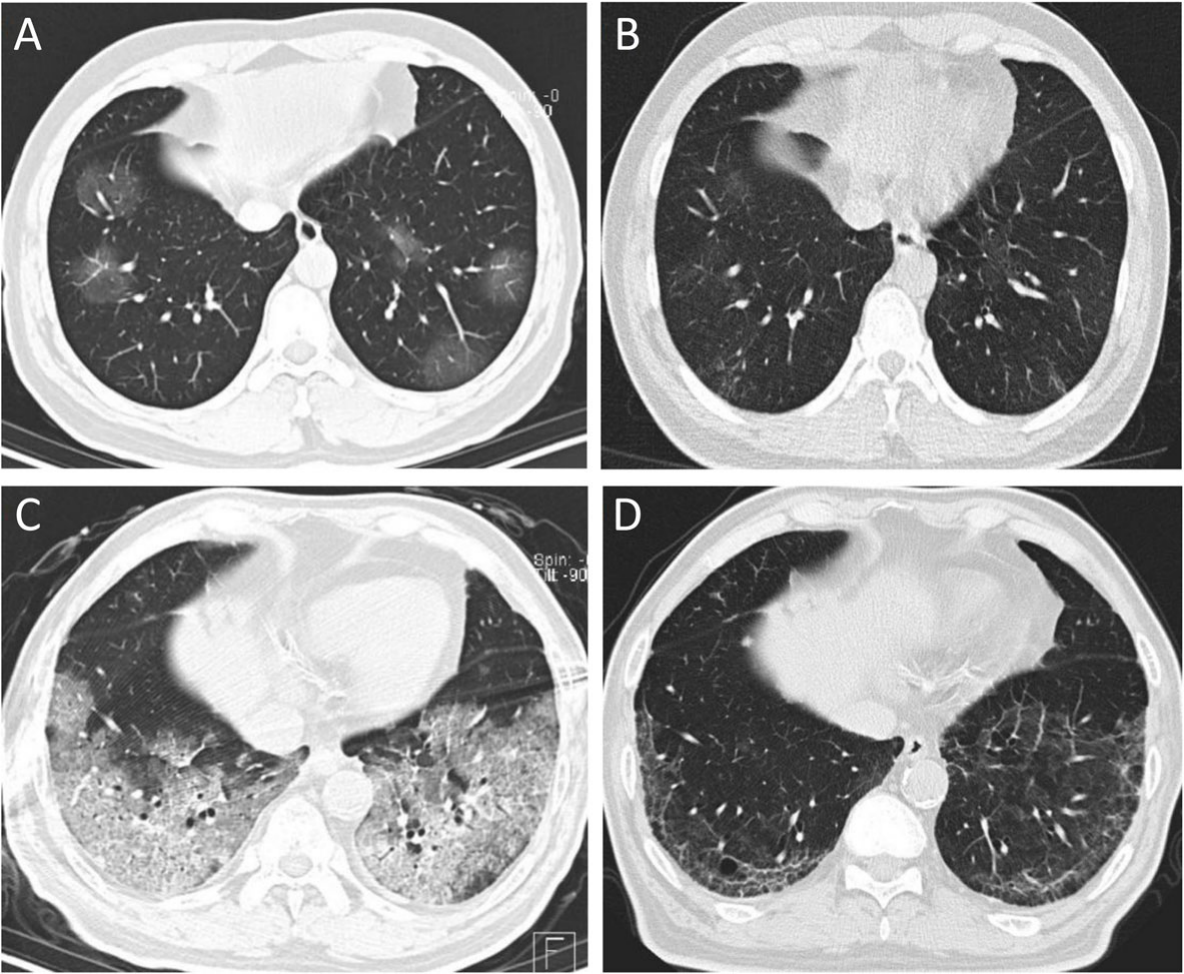
HRCT scan of a 44-year-old man in acute stage demonstrated bilateral peripheral ground-glass opacities (GGO). Lung total severity score (TSS)was 7. **B.** Follow-up CT of the same patient at 30 days after discharge from hospital showed that patchy ground glass opacity had obvious absorption.TSS was 3 .**C.** Worst CT scan of a severe patient during acute stage showed diffuse GGO, consolidation also could be seen in some area. TSS was 13. **D:** HRCT scan of the same patient obtained 30 days after discharge showed peripheral fibrosis consists of irregular linear opacities. Concomitant presence of GGO was also visible.TSS is 5

**Fig. 3:**
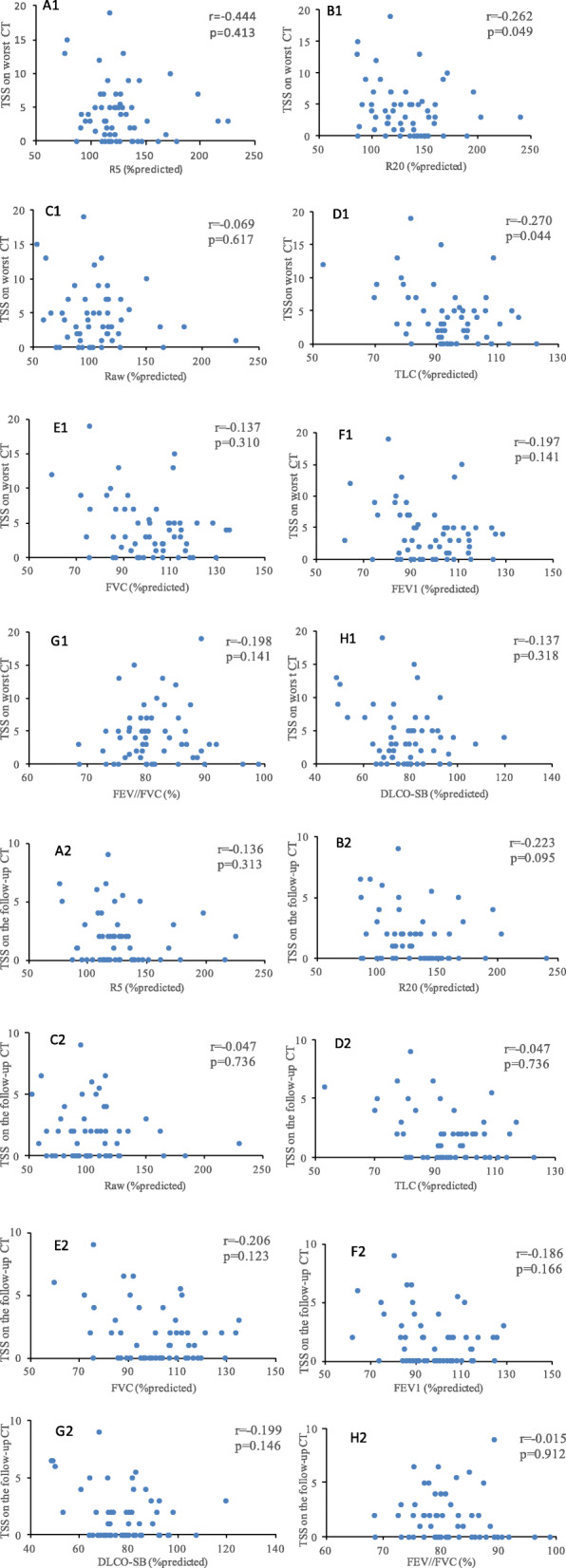
Correlation of total severity score on worst CT (from **a**1 to **h**1) and follow-up CT (from **a**2 to **h**2) with pulmonary function parameters.

During follow-up at 30 days after discharge, six patients (10.5%) complained of slight cough, four (7.0%) had shortness of breath, and three (5.3%) had occasional wheezing. Follow-up CT scan at this time showed that 31 patients (54.4%) had residual abnormality, of which 16 were severe cases (94.1%) and 15 were non-severe cases (37.5%).Most of the residual imaging abnormalities was patchy ground glass opacity with periphery distribution, which had obvious absorption compared with the worst chest CT scan (Fig. 2.a-b). Four patients had pulmonary fibrosis (Fig. 2.c-d), all of whom were severe patients. Compared with non-severe cases, severe patients had a significantly higher CT score (3.94[SD, 2.23]; 0.83 [SD, 1.39]; *p* < 0.01). At the acute phase, lung total severity score was negatively correlated with TLC and R20 (*P* = 0.049,0.044, Fig. 3), but the correlation disappeared during follow-up period.

### 6-min walk test

The mean 6-min walking distance (6MWD) in all subjects was 561.97 m (± 45.29 m). Severe patients had a shorter 6 min walking distance than non-severe patients (517.43 m [SD, 44.55 m]; 573.52 m [SD, 38.38 m], *P* = 0.012). And the 6MWD of severe cases reached only 88.4% of the predicted values, which was significant lower than non-severe cases (*p* = 0.011, Table 2).

## Discussion

Since COVID-19 broke out worldwide over the last 6 months, the mechanism, clinical characteristics, prognosis and effective treatment of the disease had not yet been adequately elucidated despite the great efforts that had been extended. Recent research and our date showed that nearly half of the discharged patients had residual abnormality in chest CT scan [6]. Global concerns are raised regarding the assessment of the lung injury for discharged patients. This study showed that in early convalescence, approximately three-quarters of patients with COVID-19 developed pulmonary function impairment, the most common of which was impaired diffusing-capacity and the decline in FEV1/FVC ratio.

DLCO abnormalities occurred in more than half of the COVID-19 patients, the data indicated impaired diffusion pathways in the intra-alveolar. To date, no other follow-up data on lung function in patients with COVID-19 can be compared. S.A. MEO et al. reported that severe acute respiratory syndrome (SARS) and COVID-19 had similar biological and clinical characteristics [9]. Previous studies on SARS survivors showed that impaired DLCO was the most common abnormality, ranging from 15.5 to 43.6% [10–15]. Our results were consistent with them. Autopsy on patients died from COVID-19 showed different degrees of destruction in alveolar structure, and pulmonary interstitial fibrosis were observed [16, 17].. Pathological changes in lungs can explain the impaired DLCO to a certain extent. Compared with non-severe case, severe patients were more likely to have DLCO abnormalities. Surprisingly, a small percentage of patients with no residual imaging abnormalities also experienced a slight decrease in DLCO. We think that these patients might have abnormal tiny blood vessels or microthrombus formation. Long-term follow-up studies of SARS survivors had shown that DLCO might remain abnormal within 3 years of recovery in some patients [18]. We will continue to perform long-term follow-up on these patients to see the trend of DLCO impairment.

Our results showed that six patients (10.5%) had obstructive pulmonary dysfunction and 7 (12.3%) had restrictive ventilation dysfunction. Two severe subjects had residual combined restrictive and obstructive type of functional impairment. Series articles on SARS survivors reporting very low rates of either obstruction or restriction, which was consistent with our research data [10, 11]. Pathological findings showed that mucous plugs were found in small airway in some severe COVID-19 patients [17], which could explain the declined ventilatory function to an extent. In addition to acute lung injury, neuromuscular weakness could also lead to decreased lung function. Certainly, a few patients suffered from lower FEV1 or FEV1/FVC ratio might due to long-term smoking or untypical airway hyperresponsiveness.

Surprisingly, during the early rehabilitation phase, lung total severity score had no significant correlation with FEV1, FVC or DLCO (Fig. 3), which was inconsistent with researches on SARS survivors [19]. It seems that the impairment of lung function was not necessarily agreed with the severity of illness or residual imaging changes. It was an interesting finding. We speculate that it was because most severe patients used glucocorticoid during hospitalization, suggesting that corticosteroids may improve the prognosis of patients with COVID-19. But most of the subjects in our hospital were imported cases from Hubei, small sample size and selection bias might affect statistical outcome. Besides, our CT quantitative evaluation was not actual represented the percentage of lung parenchyma that showed evidence of abnormalities. According to the criteria, in the same lung lobe, lung inflammatory lesions area within 25% of the difference was possible to be calculated as the same score, which might influence statistical results. In the next step, we will perform long-term follow-up study and expand the sample size to see whether similar correlation conclusions still exist.

More than half of the patients experienced a decrease in respiratory muscle strength. Approximately 29.8% of patients in our study were severe or critical, who had hypoxemia during hospitalization, requiring supplemental oxygen and bed rest, and prolonged bed rest might lead to muscle disorders. In addition, systemic use of corticosteroids might cause steroid myopathy. But when grouped by the administration of steroid, no statistical significance was found in respiratory muscle strength between the glucocorticoid group and the regular group. This result indicated that corticosteroid was not the main cause of respiratory muscle weakness. In fact, there was no difference on declining respiratory muscle strength between severe and non-severe groups. However, the direct effect of virus on respiratory muscles needs further research.

In early convalescence, the 6MWD of the severe patients was significantly shorter than that of the non-severe patients, indicating that the severe patients have poor exercise tolerance. Besides the impaired TLC and worse DLCO in severe group, we should also pay attention to cardiac function of the patients. Exercise cardiopulmonary function should be performed in further studies. Series articles on SARS survivors showed the impaired lung function existed till 1 year [10, 11]. Longer follow-up on COVID-19 patients should be made to observe the characteristic and change tendency of lung function and exercise tolerance.

There are several limitations to this study. Firstly, this is a cross-sectional study with small sample size in stratified analysis, only provides a short follow-up. The heterogeneity of our findings is not comprehensive. Secondly, only 57 of 102 COVID-19 patients (56%) in our hospital had completed the serial assessments, and the results might not be representative of the entire group. Lastly, although full lung function tests and 6MWT were conducted in our patients, we did not perform cardiopulmonary exercise testing, as many patients complained of generalized muscle weakness on follow-up. Meanwhile, labor intensity of CPET might be too high for patients in early recovery period.

In conclusion, impaired diffusing-capacity, respiratory muscle strength decrease, and lung imaging abnormalities were detected in more than half of the COVID-19 patients in early convalescence phase. Compared with non-severe cases, severe patients had a higher incidence of DLCO impairment and encountered more TLC decrease and 6MWD decline. Longer follow-up studies in COVID-19 patients should be performed to investigate the clinical outcome of recovered COVID-19 patients.

## Data Availability

All data generated or analysed during this study are included in this published article.

## Abbreviation

BMI: Body mass index
CK: Creatine kinase
CRP: C-reactive protein
WBC: White blood cell count
LDH: Lactate dehydrogenase
COVID-19: Coronavirus disease 2019
DLCO: Diffusing capacity of the lung for carbon monoxide
FVC: Forced vital capacity
FEV1: Forced expiratory volume in 1 s
LOS: Length of hospital stay
PImax: Maximum static inspiratory pressures
PEmax: Maximum static expiratory pressures
Raw: Airway resistant
R5: Airway resistance at an oscillation frequency of 5 Hz
R20: Airway resistance at an oscillation frequency of 20 Hz
TLC: Total lung capacity
TSS: Total severity score
6MWD: 6 min walk distance
6MWT: 6-min walk test
